# MALT lymphoma of the rectum, presenting with rectal prolapsus: a case report

**DOI:** 10.1186/1757-1626-3-33

**Published:** 2010-01-21

**Authors:** Feyzullah Ersoz, Ahmet Burak Toros, Hasan Bektas, Ozhan Ozcan, Oguz Koc, Soykan Arikan

**Affiliations:** 1 Department of General Surgery, Istanbul Education and Research Hospital, Istanbul, Turkey; 2 Department of Gastroenterology, Istanbul Education and Research Hospital, Istanbul, Turkey

## Abstract

Up to now, there have been only a few reported cases of Mucosa-associated lymphoid tissue (MALT) lymphomas arising in the rectum. Its clinical presentation is indistinguishable from that of rectal carcinoma but the treatment is apparently different. Symptoms of primary lymphomas involving the rectum include; anorexia, weight loss, change in bowel habits, obstruction, and bleeding. These symptoms are not disease specific and can be seen in many other gastrointestinal disorders. Patients with polypoid masses may present with obstruction symptoms. In this rare case, a female patient admitted to the emergency service with prolapsus of a rectal mass.

The optimal treatment of rectal MALT lymphoma is not well defined yet, given the rarity of the disease. Surgical resection of the localized lesion and following adjuvant chemotherapy has proved to be an effective treatment option. However, a close and long-lasting follow-up is important.

## Introduction

Mucosa-associated lymphoid tissue (MALT) lymphoma is an extranodal marginal zone B-cell neoplasm. MALT lymphoma accounts for one case in twenty cases of lymphoma. Stomach is the most common site [1]. Its development is thought to be closely associated with *H. pylori *infection. Rectum is an uncommon site for MALT lymphomas to develop, comprimising less than one percent of all colorectal malignancies [2].

It remains still unclear, whether rectal MALT lymphoma is related to H. pylori infection or not. The optimal treatment of rectal MALT lymphoma is not well defined.

Therefore we decided to report here a 44-year-old Turkish woman who admitted to the emergency service with a mass, prolapsing from the anus and to discuss the treatment options with a brief review of the literature.

## Case report

A 44-year-old Turkish woman admitted to the emergency service on March 2007, with a mass, prolapsing from the anus. After sedating the patient, we reducted the mass back into the rectum. Routine laboratory examination results were within normal ranges.

She had been smoking one packet of cigarette daily for 15 years and was not addicted to alcohol. Her anamnesis also revealed that; she couldn't defecate without digital help, since 6 months. Colonoscopy demonstrated a 7 × 7 cm sized, round and well-defined mass; covered with granular mucosa, at the lower rectum.

Histopathological examination of the biopsy specimens from the rectal lesion demonstrated a low-grade B cell lymphoma of MALT type (Extranodal marginal zone lymphoma; diffuse lymphocytic infiltrate in the lamina propria, with uniform membranous staining of the cells with the B-cell marker CD20). CD5, CD10, CD23 and bcl-6 markers were found negative. IgM and bcl-2 were found positive. Histologic examination results of the biopsy specimens from the rectal mucosa were negative for *H. pylori *infection.

The urea breath test and serum *H. pylori*-IgG antibody were all negative. We did not palpate any superficial lymph node on physical examination. Pelvic computed tomography (CT) showed an intrapelvic mass in the rectum, measuring 7 × 6 × 6 cm.

Thoracal and upper abdominal CT scans and the whole abdomen ultrasonography were reported as normal. Upper gastrointestinal endoscopy revealed normal findings. Whole-body positron emission tomographic scan and bone marrow biopsy had negative results for the disease. The patient was diagnosed as having MALT lymphoma. Her past history was unremarkable.

**Figure 1:**
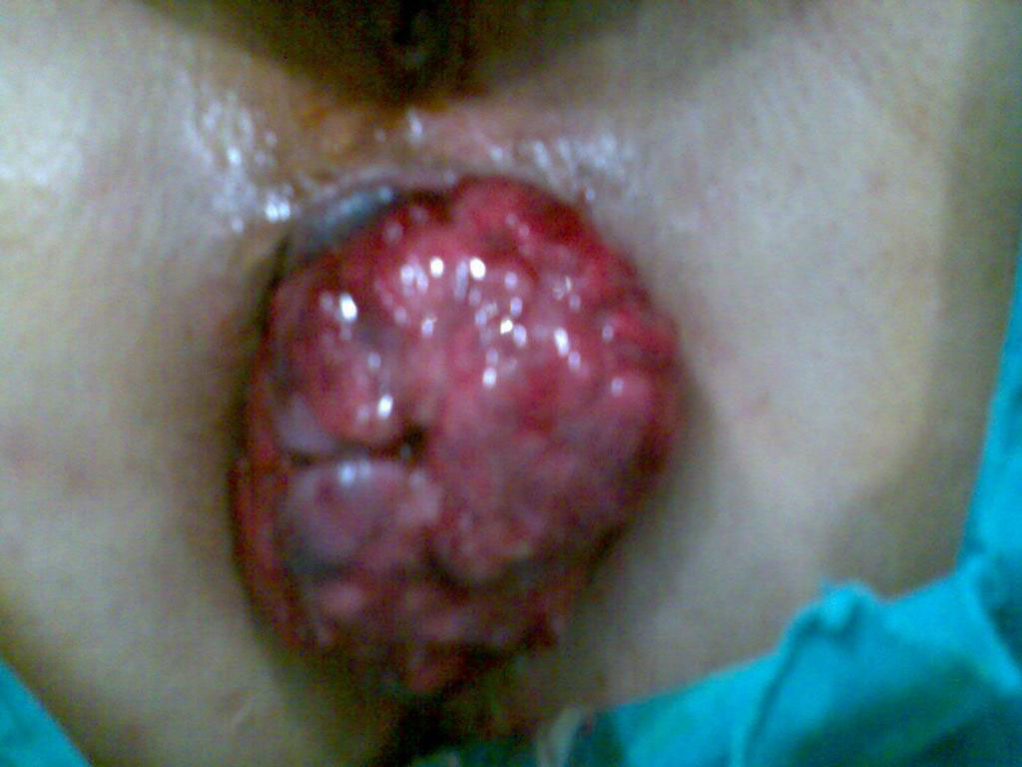
**View of the mass on the operation table**.

**Figure 2:**
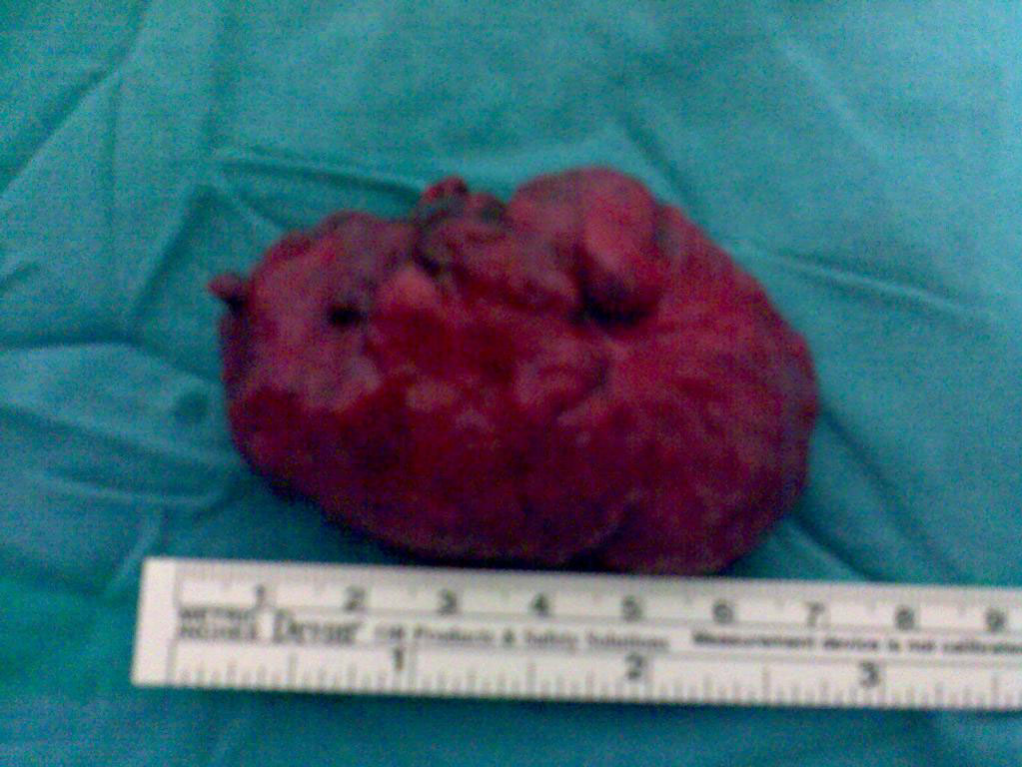
**View of the resected mass**.

The patient used to make digital interventions for defecation and the mass could get out of the anus again so we decided to make local resection of the mass under general anesthesia. The mass was excised transanally (Fig 1) (in pieces, because of friability); the tumor measured 5 × 7 cm, localized 1-2 cm proximal to the anal verge (Fig 2). After surgery, the patient received adjuvant chemotherapy (cyclophosphamide, doxorubicin, oncovin and prednisone).

Three months after the completion of therapy, a follow-up colonoscopy revealed normal mucosal view. No recurrence was detected with endoscopy and biopsies during the 27-month follow up period.

## Discussion

MALT lymphomas were first described in 1983 and since then, they are being more frequently diagnosed as a distinct clinical and pathological entity [3].

Extranodal marginal zone B-cell (MALT) lymphomas account for approximately 7.6% of all the Non-Hodgkin lymphomas; among which, they are the third most frequent histologic subtype (after the diffuse large B-cell lymphoma and the follicular lymphoma) [4]. It can be seen at any age from early adulthood to older ages, but is more common in people over the age 55. The disease is slightly more common in women than in men. MALT lymphoma is a low-grade lymphoma and often performs a slow developmental course.

The origin of rectal MALT lymphomas, is unknown. Many patients have a history of autoimmune disease such as Sjögren's syndrome or Hashimoto's thyroiditis, or bacterial infection of the stomach with *Helicobacter pylori*; our patient had none of these [5-7].

*H. pylori *infection has an important role in the development of gastric MALT lymphoma. The birth of MALT lymphoma in the stomach is preceeded with the onset of a mucosa-associated lymphoid tissue (MALT); which generally develops as the result of chronic infection by *H. pylori *[8,9].

The eradication of *H. pylori *with combination therapy, can be effectively used as the initial treatment of localized gastric MALT lymphoma. However, eradication therapy is not effective in 20-40% of cases because an AP12-MALT1 gene-positive case will not respond to eradication therapy [10]. Molecular follow-up studies revealed the persistence of the malignant clone in more than 50% of the cases with histological remission, after the antibiotic therapy. The clinical significance of this data is still unclear. Transient and self-limiting histological and molecular relapses can also happen. Therefore, a long-term follow-up is mandatory for all the patients who have received combination therapy for *H. Pylori *eradication [11].

However there are only a few reported cases of MALT lymphomas arising in the rectum. That is why the therapy options are not as clear as gastric MALT lymphomas.

Current therapeutic options for rectal MALT lymphomas include; surgical resection, endoscopic mucosal resection (EMR), different chemotherapy protocols and H. pylori eradication [12]. Antibiotics and surgery may be effective for the limited stage, low-grade MALT lymphomas [13]. When the case is not a candidate for surgery; systemic chemotherapy and radiotherapy are useful alternatives [14].

In literature, some authors have given HP eradication therapy regardless of HP positivity or negativity. Inoue et al [15], Nakase et al [16] have reported regression of MALT lymphoma of the rectum after *H. Pylori eradication *therapy; containing proton pump inhibitor, amoxicillin, and clarithromycin in patients negative for *H. pylori *infection. Grunberger et al. [17] examined the *H. pylori*-positivity rate among MALT lymphomas except for primary gastric MALT lymphomas; and found the *H. pylori*-positivity rate to be 45% (35 of 77 cases). Eradication therapy was applied to 16 *H. pylori*-positive cases. Eradication therapy was effective for only one case and there was no response at the remaining 15 cases.

Therefore, future studies on the efficacy of *H. Pylori *eradication therapy for non-gastric MALT lymphomas are warranted.

That is why we performed transanal local resection of the mass under general anesthesia.

Rectal MALT lymphoma has not been well investigated when compared to gastric MALT lymphoma. The metastatic ability of the rectal MALT lymphoma and its sensitivity to chemotherapy, is not known. For rectal MALT lymphomas, literature suggests that local surgical resection of the lesion may be the best choice [18]. For our case, surgical resection of the localized lesion and post-surgical adjuvant chemotherapy (cyclophosphamide, doxorubicin, oncovin and prednisone) were effective. Using a combination of surgery and adjuvant chemotherapy; the 5-year survival rates have increased from 27% to 55% [19]. Therefore, a long-term follow-up is essential. Follow-up at the 27 th month has revealed no evidence of tumor recurrence.

In summary; *H. pylori *infection may not have a role in the development of rectal MALT lymphoma. This patient has led us to the opinion that; in cases of localized rectal MALT lymphomas, the first step of treatment should be surgical resection and the second step is adjuvant chemotherapy. On the other hand, it is not possible to make a eligible conclusion with one case; so these patients should be followed-up for life, until more precise knowledge.

## Consent

Written informed consent was obtained from the patient for publication of this case report and accompanying images. A copy of the written consent is available for review by the Editor-in-Chief of this journal.

## Competing interests

The authors declare that they have no competing interests.

## Authors' contributions

FE, HB and OK performed the operation and collected related data. OO and SA analyzed and interpreted the patient data, gave supervision on this topic. FE and ABT designed the main text; ABT revised the article and was the major intellectual contributer. All authors read and approved the final manuscript.
